# Effect of Chromium on Mechanical Properties and Corrosion Behavior of Copper–Nickel Alloy

**DOI:** 10.3390/ma18081799

**Published:** 2025-04-15

**Authors:** Hao Chu, Zhen Yang, Yicheng Cao, Wenjing Zhang, Haofeng Xie, Yi Yuan, Hongqian Wang, Dongyan Yue

**Affiliations:** 1 State Key Laboratory of Nonferrous Structural Materials, China GRINM Group Co., Ltd., Beijing 100088, China; mse.frozen@gmail.com (H.C.); yangzhen@grinm.com (Z.Y.); caoyicheng1995@163.com (Y.C.); 18975151855@163.com (Y.Y.); hxixi719@126.com (H.W.); willimyue@foxmail.com (D.Y.); 2 GRIMAT Engineering Institute Co., Ltd., Beijing 101407, China; 3 General Research Institute for Nonferrous Metals, Beijing 100088, China

**Keywords:** copper–nickel alloy, Cr addition, erosion–corrosion

## Abstract

Copper–nickel alloys are widely applied in marine engineering due to their excellent mechanical properties and corrosion resistance. This study investigates the effect of chromium (Cr) on the erosion–corrosion behavior of copper–nickel alloys through erosion–corrosion experiments. The results indicate that the alloy containing Cr exhibits enhanced erosion–corrosion resistance. The addition of Cr reduces the corrosion rate of the copper–nickel alloy from 0.206 mm/a to 0.060 mm/a. This improvement in corrosion resistance is mainly attributed to the formation of (Ni/Fe)_3_Cr phases, which enhance the alloy’s strength and further improve its erosion resistance. Furthermore, Cr increases the electrochemical resistance of the corrosion products.

## 1. Introduction

Seawater cooling systems in ships, offshore platforms, coastal power plants, and desalination equipment face high flow-rate seawater erosion–corrosion challenges. With the development of metal materials, copper–nickel alloy gradually stood out from the many corrosion-resistant materials. Copper–nickel alloys possess excellent thermal conductivity and effectively prevent marine organism attachment, so copper–nickel alloy became the preferred choice for seawater cooling systems. However, current copper–nickel alloys exhibit limitations under extreme conditions, such as high flow rates and aggressive seawater environments. Therefore, the development of a copper–nickel alloy with better corrosion resistance is of great significance.

Copper–nickel alloy has great corrosion resistance because of its dense oxide film formed on the surface, which can separate the metal matrix from the corrosive medium. The film is mainly composed of the inner dense Cu_2_O passivation film and the loose and porous outer film, hindering the alloy from contacting the corrosive medium [1,2,3]. At the same time, the addition of Ni can also effectively fill the defects in the Cu_2_O film, further improving the corrosion resistance of Cu-Ni alloys [4,5,6].

Methods such as coating application, grain boundary engineering, and microalloying element addition are commonly used to further enhance the corrosion resistance of alloys. Typically, boundaries with Σ ≤ 29 are referred to as low-Σ CSL (Coincident Site Lattice) boundaries, also known as “special grain boundaries”, while other grain boundaries are referred to as random grain boundaries (RGB). Special grain boundaries have lower corrosion sensitivity and can effectively reduce the tendency of corrosion to propagate along grain boundaries [7,8]. Meanwhile, the addition of appropriate microalloying elements can also enhance the corrosion resistance of alloys. Based on copper–nickel alloys, the addition of Fe and Mn elements can further improve the corrosion resistance of the alloy. The addition of Fe can promote the formation of γ-FeOOH adhesion on the layer, preventing further metal ion dissolution and stabilizing the film’s amorphous structure [2,9]. However, the alloy with 1.5 wt.% Fe, after annealing at medium temperature for a long period of time, precipitates discontinuous fine Ni-Fe phases, which lead to a reduction in the corrosion resistance of the alloy [10]. Similarly, Mn can also effectively improve the corrosion resistance of copper–nickel alloys. The addition of Mn promotes the filling of cationic vacancies in corrosion products by Ni, which improves the protective ability of the corrosion product film [11]. In addition, the formation of MnO_2_ increases the diffusion resistance of Cl^−^, leading to improvement in the corrosion resistance of the alloy [12]. Other alloying elements, such as B [13], Al [14,15], and Zn [16], can also improve the corrosion resistance of Cu-Ni alloys.

The element Cr, together with Cu, Ni, Fe, and Mn, is a transition element of the fourth cycle with a similar atomic radius and electronic configurations. Due to the formation of passivation films with good protective properties, Cr is often used as a microalloying element to improve the corrosion resistance of alloys. A large number of studies have been conducted on the role of Cr in iron-based [17,18,19,20] and aluminum-based [21,22,23,24] alloys. Zhang et al. investigated the corrosion behavior of Cu-Cr and Cu-Zr alloys and found that Cr, which has a smaller atomic radius, can fill the cationic vacancies in the corrosion products, whereas Zr, which has a larger radius, cannot [25]. Xiao et al. calculated the effect of adding a third component to a Cu-10Ni alloy on the corrosive Cl^−^ adsorption energy by means of density functional theory [26]. Li et al. further investigated the static corrosion behavior of Cr-added Cu-Ni alloys in 3.5 wt.% NaCl solution [27]. However, there are few studies on the effect of Cr on the erosion–corrosion behavior and mechanism of Cu-Ni alloys. Therefore, exploring the effect of Cr on improving the erosion–corrosion resistance of Cu-Ni alloys is of great research significance.

This study designed a Cu-10Ni-1.8Fe-0.8Mn-xCr (x = 0.3, 0.4, 0.5) alloy with excellent erosion–corrosion resistance. The erosion–corrosion behavior of the new alloys and Cu-10Ni-1.8Fe-0.8Mn alloy in artificial seawater was investigated. The corrosion resistance of both alloys was measured using the weight-loss method. The alloy structure was observed using electron backscatter diffraction (EBSD) and transmission electron microscopy (TEM). The composition of the corrosion film was characterized using scanning electron microscopy (SEM) and an energy-dispersive spectrometer (EDS). The electrochemical properties of the corrosion film were analyzed using electrochemical impedance spectroscopy (EIS).

## 2. Materials and Methods

Two alloys, Cu-10Ni-1.8Fe-0.8Mn (denoted as B10) and Cu-10Ni-1.8Fe-0.8Mn-xCr (x = 0.3, 0.4, 0.5), were prepared using a vacuum melting furnace and pure copper, nickel, iron, manganese (all with 99.99 wt.% purity), and Cu-20%Cr master alloys. The chemical compositions of these alloys were measured using ICP-AES (5800 YS-HX-39, Agilent, Santa Clara, CA, USA), and the results are shown in Table 1. The deformation heat treatment process for both alloys is as follows: the cast ingot is homogenized at 975 °C for 4 h, then hot-extruded at 950 °C with an extrusion ratio of 80%. After extrusion, the rods are drawn with 15% deformation and annealed at 830 °C for 30 min.

**Figure 1:**
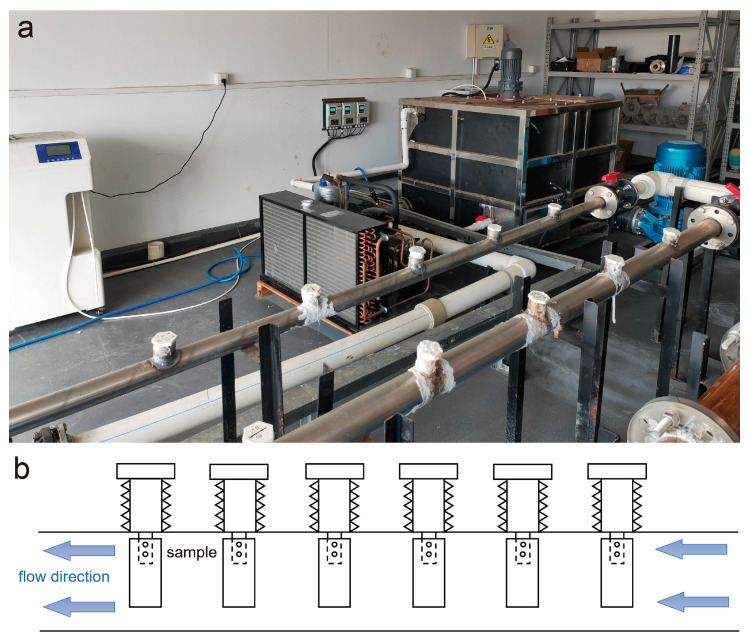
The erosion–corrosion test device: (**a**) the device in the laboratory; (**b**) the device schematic.

Samples with dimensions of 20 mm × 40 mm × 2 mm were cut from the rods, polished with sandpaper, and then subjected to erosion–corrosion experiments on a device, as shown in Figure 1. Samples are fixed in the working line by means of resin screws. The water is driven by the pump in the direction shown by the arrow, and the speed of the flow is controlled by controlling the frequency of the pump. The medium used was artificial seawater, and its composition was as follows: NaCl: 24.53 g/L; MgCl_2_: 5.200 g/L; Na_2_SO_4_: 4.090 g/L; CaCl_2_: 1.160 g/L; KCl: 0.695 g/L; NaHCO_3_: 0.201 g/L; KBr: 0.101 g/L; H_3_BO_3_: 0.027 g/L; SrCl_2_:0.025 g/L; NaF 0.003 g/L. The artificial seawater is prepared from deionized water and corresponding salt. The testing temperature was maintained at 30 ± 2 °C, with a flow rate of 3 m/s and an average dissolved oxygen content of 6.59 mg/L. The average pH during the erosion–corrosion test was 7.84. The pH was measured using a pen test pH tester (PH220, Mitutoyo, Kanagawa, Japan), and the dissolved oxygen was measured using a dissolved oxygen tester (MIK-DY2900, Asmik, Hangzhou, China). The testing period lasted for 21 days.

The samples were weighed after the erosion–corrosion test to calculate the corrosion rates. Each sample had an initial weight (W_1_) recorded before testing. After the experiment, the samples were ultrasonicated in a solution of HCl and H_2_O in a ratio of 1:1 for 3 min and then weighed a second time (W_2_). The corrosion rates were determined using the following formula:

(1) $$R=\frac{\mathrm{KW}}{\mathrm{STD}}$$

where R (mm/a) represents the corrosion rate, K is a constant = 8.76 × 10^7^, W (g) represents the mass loss (W_2_ − W_1_), S (cm^2^) represents the surface area of the sample, T (h) represents the corrosion time, and D (kg/m^3^) represents the density of the alloy.

An electrochemical workstation (VersaSTAT4, AMETEK, Berwyn, PA, USA) was used to conduct electrochemical impedance spectroscopy (EIS) and Mott–Schottky (M-S) tests. In the experiment, an Ag/AgCl electrode was used as the reference electrode, a Pt electrode was used as the auxiliary electrode, and the corroded specimen was the working electrode, with a 1 cm^2^ test area exposed. The EIS test commenced once the open circuit potential (OCP) stabilized, utilizing a frequency range of 100,000 to 0.01 Hz. The experimental results were analyzed using ZSimpWin (version 1.0.0.0) software. The microstructures of the samples in different states were analyzed via EBSD analysis using a scanning electron microscope (SEM, JSM-F100, JEOL Corp., Tokyo, Japan) and Field-emission Transmission Electron Microscope (TEM, Titan Cube, FEI Company, Hillsboro, OR, USA). Observation of the cross-section needs to be cut through the specimen by means of wire cutting, the use of epoxy resin to encapsulate the specimen to make samples, and grinding and polishing of the sample on the machine after observation. The surface morphology of the corrosion product film formed on the samples was observed using a scanning electron microscope (SEM, JEOL JSM-F100, Japan).

## 3. Results and Discussion

### 3.1. Alloy Performance Tests

**Figure 2:**
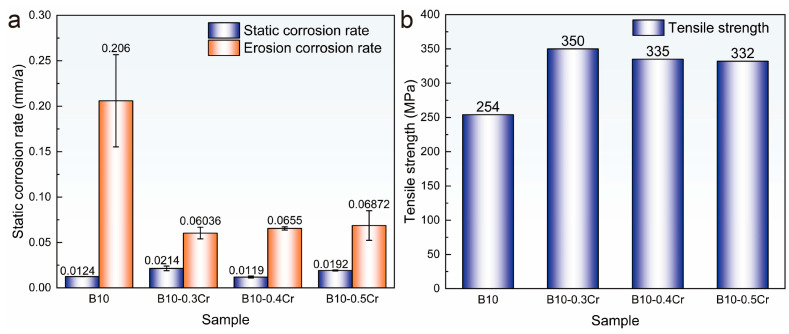
Corrosion and mechanical testing: (**a**) corrosion rate; (**b**) tensile strength.

Figure 2a shows a comparison of the corrosion rates of the four alloys. Although the static corrosion rate of the B10-xCr alloy is marginally higher than that of the B10 alloy, its erosion–corrosion resistance improves significantly, with the corrosion rate reduced by approximately 70%, showcasing its superior performance. Figure 2b compares the tensile strength of the four alloys, showing that the addition of Cr increases the strength of the B10-Cr alloy by approximately 30%. This enhancement improves impact resistance and further strengthens its erosion–corrosion performance. Among the three newly prepared alloys, the B10-0.3Cr alloy exhibits the highest corrosion resistance. Therefore, the subsequent characterization and discussion are based on the B10-0.3Cr alloy, abbreviated as (B10-Cr).

### 3.2. Microstructure of the Alloy

**Figure 3:**
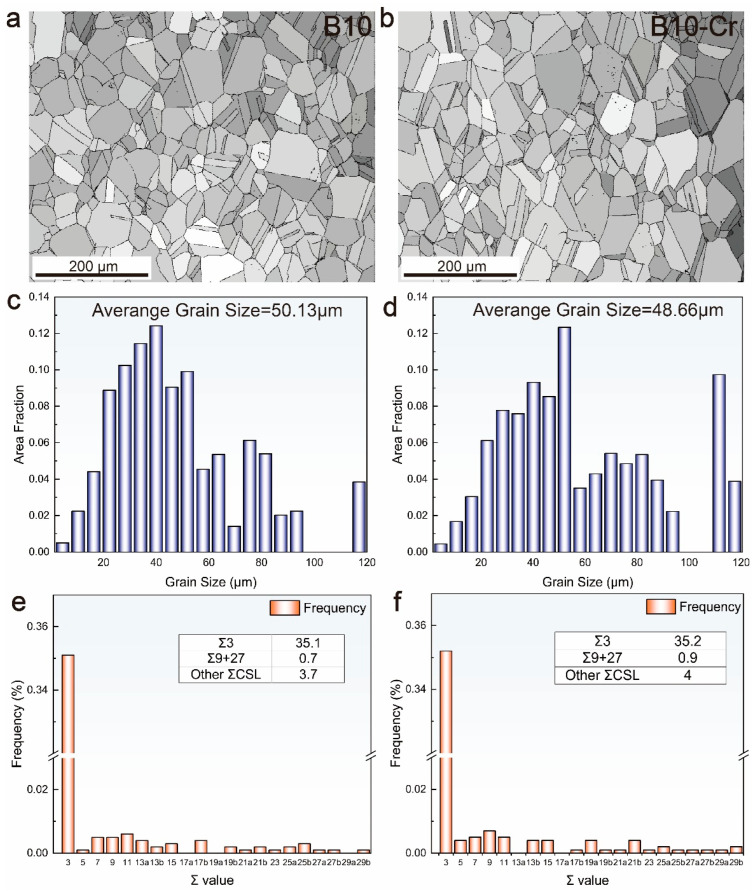
IPF images and grain size statistics of B10 and B10-Cr alloys: (**a**) IPF image of B10; (**b**) IPF image of B10-Cr; (**c**) grain size statistics of B10; (**d**) grain size statistics of B10-Cr (**e**) special boundary frequency of B10; (**f**) special boundary frequency of B10-Cr.

It is well known that grain size has a significant impact on the strength of alloys. Additionally, numerous studies have indicated that the grain size also affects the corrosion resistance of alloys [28,29,30,31]. To investigate the effect of Cr addition on the microstructure of the alloy, EBSD tests were performed on B10 and B10-Cr alloys, and the results are shown in Figure 3. In Figure 3a,b, it can be seen that the grain size distribution of the alloys is relatively uniform. In the test area, the average grain size of the B10 alloy is 50.13 μm, and the average grain size of the B10-Cr alloy is 48.66 μm, indicating that the grain sizes of the two alloys are almost identical.

At the same time, special grain boundaries can also affect the corrosion resistance of alloys [2,7]. Within the Coincident Site Lattice (CSL) model, these “special grain boundaries”, which differ from random grain boundaries in terms of their properties, have a higher degree of structural order, smaller free volume, and lower interface energy. These characteristics contribute to a stronger resistance to intergranular fracture. Among them, Σ3 is a regular twin boundary, and Σ3^n^ represents high-order twin boundaries. We statistically analyzed the proportion of special grain boundaries in both alloys, and the results are shown in Figure 3e,f. It can be seen that the distribution of special grain boundaries in both alloys is almost identical, indicating that the difference in performance between the two alloys must be due to other factors. 

**Figure 4:**
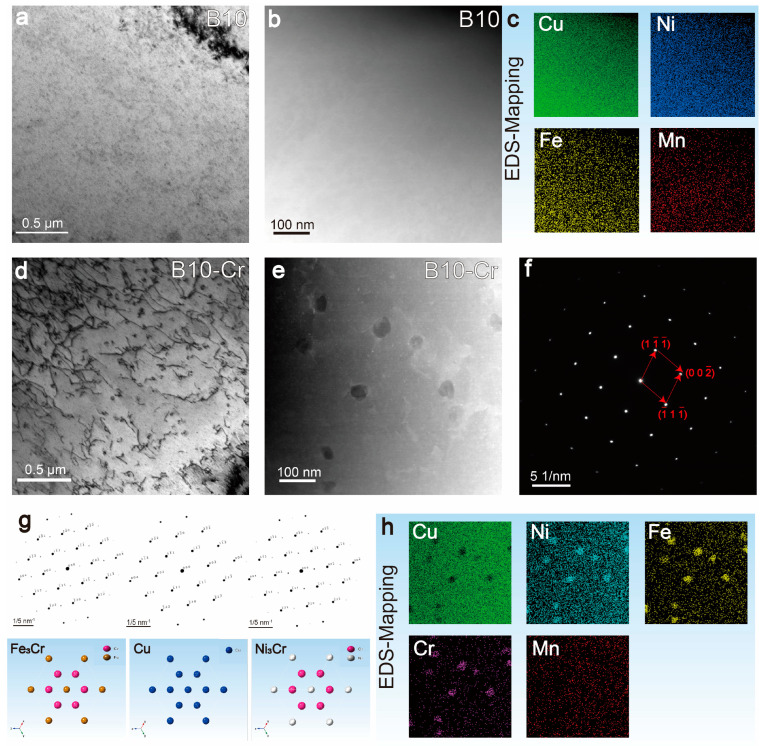
TEM images of B10 and B10-Cr alloy: (**a**) bright field of B10 alloy; (**b**) HAADF-STEM images of B10-Cr alloy; (**c**) EDS mapping of this area; (**d**) bright field of B10-Cr alloy; (**e**) HAADF-STEM images of B10-Cr alloy; (**f**) SAED of the phase; (**g**) simulated diffraction spots of (Fe/Ni)_3_Cr and Cu on [110] direction; (**h**) EDS mapping of this area.

To investigate the microscopic changes that contributed to the improvement in mechanical and corrosion properties, TEM analysis was conducted on both the B10 and B10-Cr alloys, as shown in Figure 4. After cold deformation and annealing, the B10 alloy remained in a single-phase state. In the B10-Cr alloy, no second phase with clear contrast was observed in the bright-field image. However, in the HAADF-STEM mode, a second phase rich in Cr, Ni, and Fe was found at dislocation entanglements, with a size of approximately 10–20 nm, as shown in Figure 4d–f. The diffraction behavior of this phase was almost identical to that of the copper matrix, making it difficult to detect in the bright-field image, but it was more prominent in the HAADF-STEM mode. We attempted to obtain selected electron diffraction images of this phase, but due to the small particle size and low concentration of this phase, no superlattice diffraction peaks were observed in the diffraction spectrum.

The fine, dispersed second phase significantly enhances the strength of the matrix, which is the main reason for improving its mechanical properties and greatly increasing the alloy’s resistance to erosion. However, at the same time, the presence of the second phase creates a potential difference between the phase and the matrix, which can cause selective phase corrosion to some extent, leading to a slightly higher static corrosion rate in the B10-Cr alloy compared to the B10 alloy. Combining the compositional and diffraction pattern information in Figure 4f,h, it can be inferred that the phase is (Ni/Fe)_3_Cr with an L12 structure. The Ni_3_Cr and Fe_3_Cr phases have the same lattice type and similar lattice constants (Ni_3_Cr: a = b = c = 3.5446 Å; Fe_3_Cr: a = b = c = 3.5640 Å), where Ni and Fe atoms are interchangeable. The simulated diffraction spots of the two phases and Cu matrix in the [110] direction are shown in Figure 4g.

### 3.3. Microstructure of Corrosion Product Film

**Figure 5:**
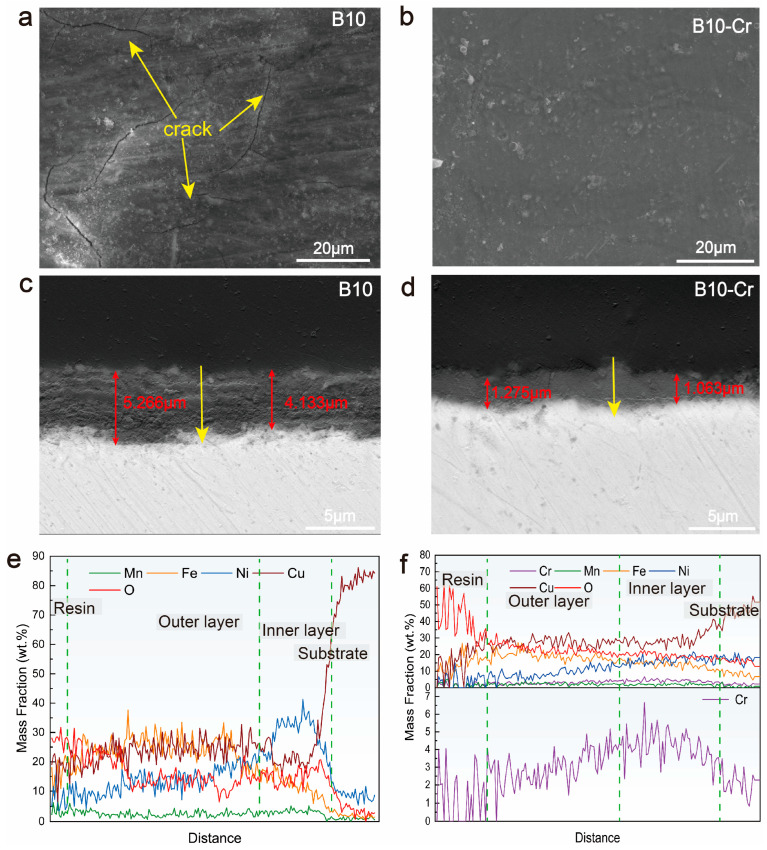
Microstructure of corrosion product film: surface of the corrosion product film: (**a**) B10; (**b**) B10-Cr; cross-sections of the corrosion product film: (**c**) B10; (**d**) B10-Cr; line scan results: (**e**) B10; (**f**) B10-Cr.

Figure 5a,b show the SEM images of the corrosion product film surface of the two alloys. After 21 days of erosion–corrosion, the corrosion product surface of B10 alloy appears to have more obvious cracks. However, the surface of the corrosion products of B10-Cr alloy was relatively smooth and flat, without obvious cracks and detachment, indicating that the corrosion products were tightly bonded with the matrix.

Many studies have indicated that the corrosion products of copper–nickel alloys exhibit a multi-layered film structure [2,7,11,32]. To study the impact of Cr addition on the corrosion product film structure, the corrosion product cross-section was analyzed using SEM, as shown in Figure 5c–f. Figure 5c,d show that the corrosion product film on the B10 alloy is about 4–5 μm thick, while for the B10-Cr alloy, it is only about 1 μm. The addition of Cr resulted in a more compact corrosion product film, thereby enhancing its protective properties. The compositional analysis of the corrosion products was conducted along the path marked by the yellow arrow in Figure 5e,f. Compositional analysis of the corrosion products indicated that both alloys exhibited Ni enrichment near the metal substrate, forming the inner layer of the corrosion product film. In the B10-Cr alloy, Cr was also enriched in the corrosion product film, further improving the protective properties. It was pointed out that Cr^3+^ filled in the cationic vacancies of the Cu_2_O membrane due to the slightly smaller ionic radius of Cr^3+^ compared to Cu^+^, decreased the concentration of the vacancies, and increased the resistance to ionic diffusion [25]. 

Many researchers have proposed that Cu corrosion generally occurs in the presence of a Cl^−^ environment [1,33], in the form of dissolution and then precipitation to ultimately produce Cu_2_O, blocking the direct contact between the metal substrate and the corrosive medium.

Cu →Cu^+^ + e^−^ (2)

Cu^+^ + Cl^−^ → CuCl (3)

CuCl + Cl^−^ → CuCl_2_^−^ (4)

Combined with the cathodic reaction, this ultimately produces Cu_2_O:

O_2_ + 2H_2_O + 4e^−^ → 4OH^−^ (5)

2CuCl_2_^−^ + 2OH^−^ → Cu_2_O + H_2_O + 4Cl^−^ (6)

As the electronic configuration of Ni^2+^ and Cu^+^ is the same and the ionic radiuses were not much different, Ni^2+^ filled into the cationic vacancies of Cu_2_O films. When the Ni filler concentration reached a certain level, part of the Cu_2_O was transformed into amorphous NiO [32]. This explains the enrichment of Ni in corrosion products. Researchers have pointed out that the aggregation of Fe in the outer layers of the corrosion products relates to the γ-FeOOH, which accompanied CuO in the outer layer of the film. The Fe stays in the outer layer of the corrosion product film, preventing further metal ion dissolution and stabilizing the film’s amorphous structure [2].

### 3.4. Electrochemical Performance Characterization of Alloys

**Figure 6:**
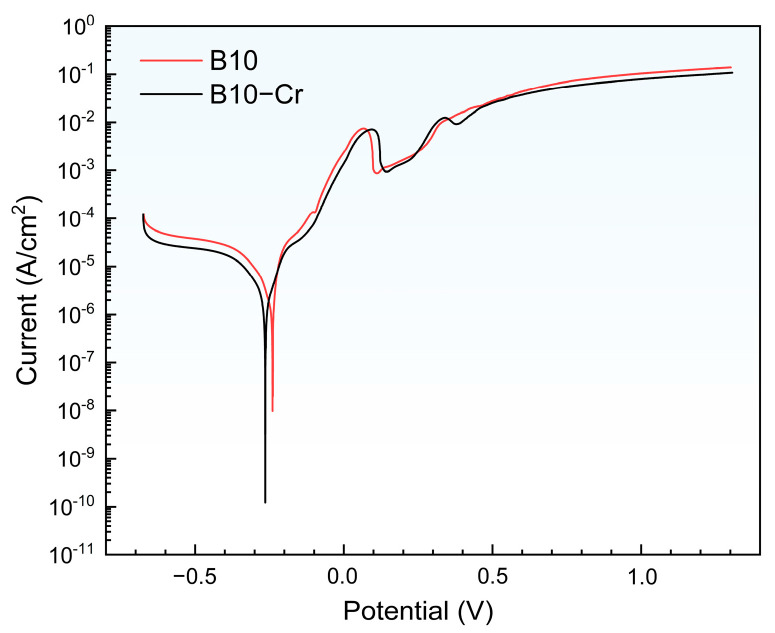
The polarization curves of two alloys with polished surface.

Figure 6 shows the polarization curves for two alloys with polished surface. In the figure, it can be seen that the corrosion potential of the B10-Cr alloy (approximately −0.26 mV) is lower than that of the B10 alloy (approximately −0.23 mV). At any given potential, the passive current density of the B10-Cr alloy is slightly lower than that of the B10 alloy. The results suggest that the B10-Cr alloy is more easily passivated than the B10 alloy, indicating that the B10-Cr alloy has better corrosion resistance. This may be because Cr is more easily passivated than Cu. The Nernst equation is as follows:

(7) $$E=E^{\theta }+\frac{\mathrm{RT}}{\mathrm{zF}}\ln\frac{\mathrm{ox}}{\mathrm{red}}$$

where E is the electrode potential in the actual state, E^θ^ is the electrode potential in the standard state, R is the ideal gas state constant, T is the temperature, z is the number of electrons transferred during the electrochemical reaction, and F is Faraday’s constant. The term “ox” and “red” represent the concentrations of the oxidizing and reducing species, respectively. Combined with the average pH in the experimental environment, the electrode potentials for the electrode reactions of several alloying elements were calculated as Cu: 0.016 V; Ni: −0.26 V; Fe: −0.50 V; Cr: −1.18 V. The lower reaction potential of Cr makes Cr easier to passivate, and thus the B10-Cr alloy has a lower corrosion potential [34,35].

**Figure 7:**
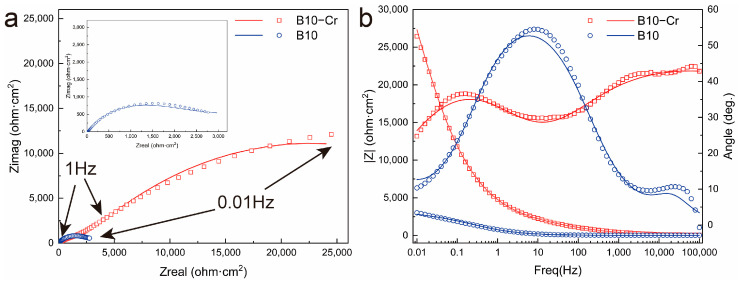
The EIS results: (**a**) Nyquist plots of B10 and B10-Cr alloy; (**b**) bode plots of the two alloys.

Figure 7a,b show the electrochemical impedance spectroscopy (EIS) results and the fitting circuit diagram for the corroded samples. In the Nyquist plot, it can be observed that after adding Cr, the capacitive arc radius significantly increases, indicating that the corrosion product film resistance of the B10-Cr alloy is much higher than that of the B10 alloy, and there may be Winberg impedance present. In the Bode plot, it can be seen that in the low-frequency region, the impedance modulus of the B10-Cr alloy is greater than that of the B10 alloy, suggesting that the corrosion film of the B10-Cr alloy has better corrosion resistance and protective ability, which also explains its superior corrosion resistance.

**Figure 8:**
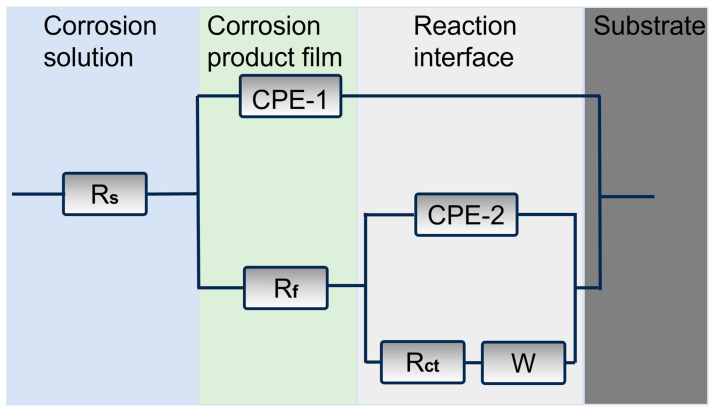
Fitting equivalent circuit of the two alloys.

In the Bode plot, there are two peaks in the phase angle as a function of frequency, which indicates that the fitting circuit should have two time constants. Since the corrosion product film has a bilayer structure, the circuit shown in Figure 8 is used for fitting, where Rs represents the solution resistance, while CPE-1and R_f_ are in parallel, CPE-1 describes the capacitance of the corrosion product film, and R_f_ describes the resistance of corrosion product film. CPE-2 describes the double layer capacitance, and R_ct_ describes the charge transfer resistance. The symbol W denotes the Warburg impedance, indicating the diffusion process. Considering the roughness of the surface of the corrosion product, the constant phase element (CPE) is used instead of the ideal capacitive element.

Table 2 presents the equivalent circuit fitting results of the electrochemical impedance spectroscopy (EIS) data, with a χ^2^ value on the order of 10^−3^, indicating good fitting quality. For the purpose of discussion, the admittance values obtained from the constant phase element fitting are considered as the capacitance values of an ideal capacitor, as shown in the table. The charge transfer resistance of B10-Cr is higher than that of B10, with the charge transfer between the electrolyte and electrode interfaces in B10-Cr alloys being more difficult, suggesting that the corrosion product film on the surface of B10-Cr provides stronger protection compared to the surface of B10. The EIS results indicate that the corrosion product film on B10-Cr has better protective properties.

## 4. Conclusions

(1) Cr has a notable synergistic effect on the erosion–corrosion resistance and strength of cupronickel alloys, with optimum alloy properties achieved with the addition of 0.3 wt.% Cr, increasing the ultimate tensile strength of cupronickel alloys by 30% to 350 MPa and reducing the erosion–corrosion rate by 70% to 0.0603 mm/a.
(2) The addition of Cr has almost no effect on the grain size and the proportion of special grain boundaries in the alloy. However, TEM results show that the addition of Cr forms fine, dispersed (Ni/Fe)_3_Cr phases in the matrix, which is the main reason for the increased strength of the alloy. At the same time, this also improves the erosion resistance of the alloy matrix.
(3) Characterization of the corrosion products revealed that the B10-Cr alloy formed smoother films with fewer cracks, and there is also an enrichment of Cr in the corrosion products. This indicates that Cr can partially fill the cation vacancies in Cu_2_O, thereby reducing the ion diffusion pathways. The results of the polarization curves show that the B10-Cr alloy is easier to passivate, which allows the alloy matrix to be protected by the passive film at an earlier stage. The EIS results show that the addition of Cr greatly improves the resistance to charge transfer, thereby improving the corrosion performance of the alloy.

In future studies, we will further investigate the precipitation behavior of finely dispersed chromium-containing phases and its effect on the corrosion resistance of the alloy.

## Figures and Tables

**Table 1:** Compounds of the alloys.

Sample	Ni (wt.%)	Fe (wt.%)	Mn (wt.%)	Cr (wt.%)	Cu (wt.%)
B10	9.98 ± 0.02	1.89 ± 0.01	0.90 ± 0.02	-	Bal.
B10-0.3Cr	9.98 ± 0.02	1.93 ± 0.01	0.90 ± 0.03	0.29 ± 0.01	Bal.
B10-0.4Cr	9.96 ± 0.02	1.93 ± 0.02	0.90 ± 0.03	0.41 ± 0.01	Bal.
B10-0.5Cr	9.98 ± 0.04	1.93 ± 0.02	0.90 ± 0.01	0.52 ± 0.02	Bal.

**Table 2:** Fitting parameters of B10 and B10-Cr after erosion–corrosion in artificial seawater for 21 days.

Sample	R_s_ (Ω·cm^2^)	C_1_ × 10^−6^ (F·cm^2^)	n_1_	R_f_ (Ω·cm^2^)	C_2_ × 10^−6^ (F·cm^−2^)	n_2_	R_ct_ (Ω·cm^2^)	W × 10^3^ (Ω^−1^·cm^−2^·s^−0.5^)	∑x^2^ × 10^−3^
B10	19.86	31.65	0.68	2211	20.59	0.82	5184	7.15	2.549
B10-Cr	1.28	27.42	0.53	2798	77.37	0.73	5.762 × 10^4^	5.139	0.523

## Data Availability

The original contributions presented in this study are included in the article. Further inquiries can be directed to the corresponding authors.

## References

[1] Kear G., Barker B.D., Walsh F.C. (2004). Electrochemical corrosion of unalloyed copper in chloride media—A critical review. Corros. Sci..

[2] Ma A.L., Jiang S.L., Zheng Y.G., Ke W. (2015). Corrosion product film formed on the 90/10 copper–nickel tube in natural seawater: Composition/structure and formation mechanism. Corros. Sci..

[3] North R.F., Pryor M.J. (1970). The influence of corrosion product structure on the corrosion rate of Cu-Ni alloys. Corros. Sci..

[4] Jin T.Z., Zhang W.F., Li N., Liu X.R., Han L., Dai W. (2019). Surface Characterization and Corrosion Behavior of 90/10 Copper-Nickel Alloy in Marine Environment. Materials.

[5] Yuan S.J., Pehkonen S.O. (2007). Surface characterization and corrosion behavior of 70/30 Cu–Ni alloy in pristine and sulfide-containing simulated seawater. Corros. Sci..

[6] Zhang Y., Song C., Cao Y., Wang H., Wang Z., Yang B. (2020). Microstructure and Corrosion Behavior of as-cast 90Cu-10Ni Alloys with Different Yttrium Content. Int. J. Electrochem. Sci..

[7] Zhao Y., Peng L., Xie H., Zhang W., Huang S., Yang Z., Li Z., Mi X. (2023). Enhancing the erosion-corrosion resistance of cupronickel alloy through grain boundary engineering. Corros. Sci..

[8] Wei X.X., Zhang B., Wu B., Wang Y.J., Tian X.H., Yang L.X., Oguzie E.E., Ma X.L. (2022). Enhanced corrosion resistance by engineering crystallography on metals. Nat. Commun..

[9] Zhu Y.B., Chen X.H., Liu P., Fu S.L., Zhou H.L., Wu J.Y. (2021). Effect of iron on the composition and structure of corrosion product film formed in 70/30 copper-nickel alloy. Anti-Corros. Methods Mater..

[10] Drolenga L.J.P., Ijsseling F.P., Kolster B.H. (1983). The Influence of Alloy Composition and Microstructure on the Corrosion Behavior of Cu-Ni Alloys in Seawater. Werkst. Und Korros.-Mater. Corros..

[11] Shao G., Gao Y., Wu J., Liu P., Zhang K., Li W., Ma F., Zhou H., Chen X. (2022). Effect of Fe/Mn content on mechanical and corrosion properties of 90/10 copper-nickel alloy. Mater. Corros.-Werkst. Korros..

[12] Zhu Z., Li S., Zhang R. (2021). Investigation of corrosion characteristics of Cu-10Ni-1.2Fe-*x*Mn (*x* = 0.53, 0.87, 1.19) alloy in 3.5% NaCl solution. RSC Adv..

[13] Dong X., Pan L., Jin Q., Wang C., Jiang Y. (2010). Effects of B on Microstructure and Properties of White Copper Alloys. Spec. Cast. Non-Ferr. Alloys.

[14] Chang Y., Hong S., Zhang S. (2023). Effect of Al Content on the Corrosion Resistance of Cu-Ni Powder Metallurgy Alloy. Mater. Prot..

[15] Song Z., Jia S., Zhou Y., Song K., Mi X., Li Z. (2018). Effect of homogenization annealing on corrosion resistance of new Cu-Ni-Al alloy. Trans. Mater. Heat Treat..

[16] Tandon V., Patil A.P., Bansod A. (2018). Effect of Zn addition on corrosion resistance of Cu-10Ni alloy in clean and sulphide contaminated seawater. Can. Metall. Q..

[17] Sun J., Tang H., Wang C., Han Z., Li S. (2022). Effects of Alloying Elements and Microstructure on Stainless Steel Corrosion: A Review. Steel Res. Int..

[18] Cui J., Zhang Y., Wang L., Chen X., Zhang G. (2014). Chemical Composition Optimization and Sea Water Corrosion Resistance of a Ductile Cast Iron. J. Chin. Soc. Corros. Prot..

[19] Tomaru M., Yakou T. (2011). Effect of Cr Contents on Corrosion Resistance of FeAl in HCl Solution. Tetsu Hagane-J. Iron Steel Inst. Jpn..

[20] Wang F., Shu Y. (2003). Influence of Cr content on the corrosion of Fe-Cr alloys: The synergistic effect of NaCl and water vapor. Oxid. Met..

[21] Esquivel J., Gupta R.K. (2020). Review—Corrosion-Resistant Metastable Al Alloys: An Overview of Corrosion Mechanisms. J. Electrochem. Soc..

[22] Fang H.C., Chao H., Chen K.H. (2014). Effect of Zr, Er and Cr additions on microstructures and properties of Al-Zn-Mg-Cu alloys. Mater. Sci. Eng. A-Struct. Mater. Prop. Microstruct. Process..

[23] Fang H.C., Chen K.H., Chen X., Chao H., Peng G.S. (2009). Effect of Cr, Yb and Zr additions on localized corrosion of Al-Zn-Mg-Cu alloy. Corros. Sci..

[24] Moon G., Lee E.K.Y. (2024). Simultaneous refinement in 0′ precipitation and corrosion behavior of a 2xxx series Al-Cu alloys modified via the sole or joint addition of Cr, Mn and Zr. Mater. Today Commun..

[25] Zhang Y.N., Zi J.L., Zheng M.S., Zhu J.W. (2008). Corrosion behavior of copper with minor alloying addition in chloride solution. J. Alloys Compd..

[26] Xiao X., Liu X., Wang Z., Xu X., Chen M., Xie J. (2024). Corrosion mechanism and corrosion behavior prediction of Cu-10Ni-X alloys in NaCl solution combining DFT calculation and experiments. Corros. Sci..

[27] Li S., Fang M., Xiao Z., Meng X., Lei Q., Jia Y. (2023). Effect of Cr addition on corrosion behavior of cupronickel alloy in 3.5 wt% NaCl solution. J. Mater. Res. Technol..

[28] Aung N.N., Zhou W. (2010). Effect of grain size and twins on corrosion behaviour of AZ31B magnesium alloy. Corros. Sci..

[29] Gollapudi S. (2012). Grain size distribution effects on the corrosion behaviour of materials. Corros. Sci..

[30] Ralston K.D., Fabijanic D., Birbilis N. (2011). Effect of grain size on corrosion of high purity aluminium. Electrochim. Acta.

[31] Wang B., Liu Q., Wang X. (2012). Effect of Grain Size on Atmospheric Corrosion Resistance of Ultra-Low Carbon If Steel. Acta Metall. Sin..

[32] Wu L., Xu Y., Ma A., Zhang L., Zheng Y. (2024). Influence of pre-immersion aeration conditions on corrosion product films and erosion-corrosion resistance of 90/10 and 70/30 copper-nickel tubes in 1 wt% NaCl solution. Corros. Sci..

[33] Campbell S.A., Radford G.J.W., Tuck C.D.S., Barker B.D. (2002). Corrosion and Galvanic Compatibility Studies of a High-Strength Copper-Nickel Alloy. Corrosion.

[34] Ura-Binczyk E., Homazava N., Ulrich A., Hauert R., Lewandowska M., Kurzydlowski K.J., Schmutz P. (2011). Passivation of Al–Cr–Fe and Al–Cu–Fe–Cr complex metallic alloys in 1M H2SO4 and 1M NaOH solutions. Corros. Sci..

[35] Zhu M., Li K., Liu Y., Wang Z., Yao L., Fa Y., Jian Z. (2020). Microstructure, Corrosion Behaviour and Microhardness of Non-equiatomic Fe_1.5_CoNiCrCux (0.5 ≤ x ≤ 2.0) High-Entropy Alloys. Trans. Indian Inst. Met..

